# Crossing the Boundaries of Our Current Healthcare System by Integrating Ultra-Weak Photon Emissions with Metabolomics

**DOI:** 10.3389/fphys.2016.00611

**Published:** 2016-12-15

**Authors:** Rosilene C. Rossetto Burgos, Eduard P. A. van Wijk, Roeland van Wijk, Min He, Jan van der Greef

**Affiliations:** ^1^Division of Analytical Biosciences, Leiden Academic Center for Drug Research, Leiden UniversityLeiden, Netherlands; ^2^Sino-Dutch Center for Preventive and Personalized Medicine/Center for Photonics of Living Systems, Leiden UniversityLeiden, Netherlands; ^3^Meluna Research in BiophotonicsGeldermalsen, Netherlands

**Keywords:** ultra-weak photon emission, metabolomics, healthcare, system biology, diagnostics

## Abstract

The current healthcare system is hampered by a reductionist approach in which diagnostics and interventions focus on a specific target, resulting in medicines that center on generic, static phenomena while excluding inherent dynamic nature of biological processes, let alone psychosocial parameters. In this essay, we present some limitations of the current healthcare system and introduce the novel and potential approach of combining ultra-weak photon emission (UPE) with metabolomics technology in order to provide a dynamic readout of higher organizational systems. We argue that the combination of metabolomics and UPE can bring a new, broader, view of health state and can potentially help to shift healthcare toward more personalized approach that improves patient well-being.

## Introduction

There is currently a global need for a change in the healthcare system, including healthcare policies. Specifically, we have clearly reached the boundaries of our current system, and rising costs make healthcare increasingly less affordable.

The past success of the healthcare system is based on a paradigm that—ironically—is now a limitation. The discovery of penicillin in 1928 by Alexander Fleming started an era of antibiotic development and laid the foundation for a “war against” system of healthcare. Over the past century, this concept became increasingly popular and has been particularly successful for treating acute illnesses. This paradigm is exemplified by terms commonly used in modern medicine with prefixes and suffixes such as “anti-” (e.g., antibiotic), “-inhibitor” (e.g., angiotensin-converting-enzyme inhibitor—ACE), and “-blocker” (e.g., beta-blocker). This approach led to the development of a disease management system rather than a bona fide healthcare system. Most importantly, this system largely overlooks chronic illness and preventive strategies (Choi et al., 2005; Hunter, 2008).

Rather than extrapolating current disease management knowledge into disease prevention and the promotion of health—a strategy that is likely to fail—we suggest that a radically different, transformative approach is needed. This novel approach is based on a systems—or ecological—view on health and well-being (Layard, 2005; Puska, 2008). With this approach, the focus shifts naturally from a more reductionist, single-symptom approach to a dynamic perspective based on systems regulation. In addition, each individual patient is considered in context, providing a more biopsychosocial view rather than a strict biomedical perspective. Such a shift from the traditional “one-size-fits-all” approach—which typically leads to a “one-size-fits-none” outcome—to personalized medicine/healthcare is a central theme in the new paradigm referred to as “P4 Health”: Preventive, Predictive, Personalized, Participatory healthcare (Hood and Friend, 2011).

In a health ecosystem, human-human interactions are the most important basis, with compassion and respect for each other's world view serving as the central theme (van der Greef et al., 2010). Moreover, politicians and policy-makers must change their focus from a short-term “quick-fix” approach, which is typically driven by short-term electoral cycles, to achieving long-term, sustainable improvements in our healthcare system at the societal level. The drastic, wide-reaching effects of our changing lifestyle on health and well-being—which has created the so-called “diseases of comfort”—must be considered from a much wider perspective, requiring an approach that crosses cultures as well as disciplines. This approach will improve integrative medicine and facilitate health-focused prevention, thereby reaffirming the importance of the relationship between the physician and patient by focusing on the whole person; moreover, this approach will be evidence-based and will integrate all appropriate therapeutic and lifestyle strategies, healthcare system, and disciplines in order to achieve optimal health and healing (Academic Consortium for Integrative Medicine & Health, 2016).

From the perspective of diagnostics, current approaches focus only on a single time point or a limited number of time points; however, human physiology is based on a wide spectrum of endogenous biological rhythms and oscillations (Muehsam and Ventura, 2014). Such rhythms serve as a fingerprint representing higher-order dynamic systems and various time scales ranging from long periods such as diurnal/nocturnal, monthly, seasonal, and annual rhythms to short periods on the order of minutes, seconds, or fractions of seconds. In this context, human physiology tends to maintain a homeostatic state due to a complex network of regulatory feedback circuits driven by various rhythms. Therefore, homeostasis and allostasis go hand-in-hand with dynamic systems concepts (Sterling, 2012; van der Greef et al., 2013), and changes in oscillation patterns and/or rhythms can indicate a perturbation in the system. Indeed, evidence suggests that examining one's biological clock might help with determining a clinical diagnosis (Most et al., 2010). However, current clinical biochemistry tools are limited with respect to bridging different rhythmic time scales. Therefore, methods for measuring ultra-weak photon emissions (UPE) have been developed and appear to be suitable for measuring systems dynamics. In addition, UPE can be measured in real time and is non-invasive, label-free, and cost-effective.

A growing body of evidence suggests that UPE reflects the coherence of self-organizing systems and might therefore be used to measure health at a higher organizational level (Bajpai et al., 2013; de Mello Gallep, 2014; Van Wijk et al., 2014), providing a novel tool for reading the state of dynamic biological systems. UPE is intrinsic to every living system that undergoes respiration and utilizes oxygen; therefore, the dynamics of UPE reflect the biological processes that underlie this emission (Cifra and Pospíšil, 2014; Pospíšil et al., 2014; van Wijk, 2014). We therefore suggest that UPE may be combined with metabolomics technologies in order to develop an integrated diagnostic tool for detecting the transition from health to disease by combining the sensitivity of biochemical pattern recognition with the high temporal resolution of UPE measurement.

Metabolomics has been described as the comprehensive analysis of all metabolites in a biological sample (e.g., cells, tissues, and/or bodily fluids; German et al., 2005; Ramautar et al., 2013). Together with other omics technologies (i.e., genomics, transcriptomics, and proteomics), metabolomics provides a holistic picture of the metabolic phenotype (Beger et al., 2016). The major advantage of using metabolomics over other omics technologies is the ability to investigate dynamic regulatory mechanisms at the molecular level, providing insight into how distinct biochemical pathways are interconnected (German et al., 2005). Thus, a more personalized approach to health assessment can be achieved.

**Figure 1:**
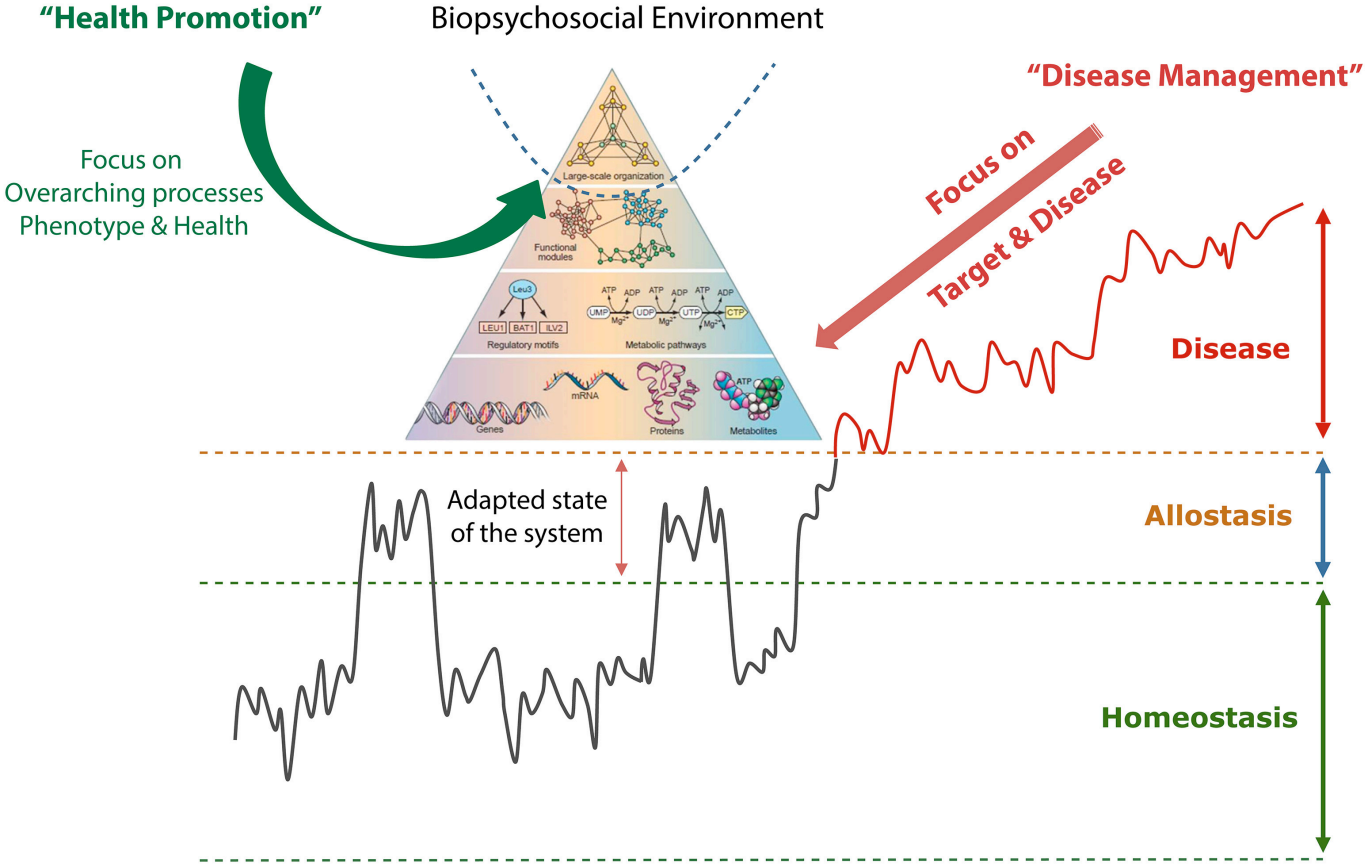
**Schematic depiction of the development from health into a disease state**. It shows how challenges to homeostasis can be regulated by allostasis by adapting the set points of the regulatory system. If the resilience is lost over time, the system can develop into a disease state. The last part is often handled by disease management focusing on single symptoms. The “life pyramid” view represents the notion of how biological systems are interconnected at different organizational levels and how the biopsychosocial environment acts dynamically within the system and therefore reflects the information at the lower levels. This integrated picture is more applicable to the pre-disease and healthy state and represents the newly emerging picture of how the future healthcare system should focus on promoting health rather than treating disease. Adapted from Oltvai and Barabási (2002), Ramautar et al. (2013), and van der Greef et al. (2013).

In addition, new ideas regarding systems-based thinking have appeared, accelerating our understanding of concepts regarding the complexities of life (Oltvai and Barabási, 2002). Nevertheless, our healthcare system's current focus on disease management using a reductionist approach does not consider influences of the biopsychosocial environment. Moreover, novel fundamental advances in our understanding of the dynamics of life have led to the development of a new biological picture. In this view, the organizational metabolic network is represented as a hierarchical model (i.e., as a pyramid; see Figure 1). Another newly recognized feature is the dynamic, oscillatory aspect of metabolic flows, in which metabolic regulatory information is controlled by repetitive, pulsing dynamic systems (Adachi et al., 1999).

In this essay, we address the potential new diagnostic tools as a stepping stone toward realizing novel intervention-based strategies in a health ecosystem based on promoting the healthy state (i.e., salutogenesis). The salutogenesis concept has been used in many health practices. The concept can be seen as complementary to the biomedical model as it focuses on the complex self-healing processes rather than concentrating on a singular pathogenic factor (Antonovsky, 1984; Lindstrom and Eriksson, 2005).

## Moving toward a new vision

Our current medical system is based on pathogenesis, defining disease, and developing standardized treatments. However, the cornerstones of the healthcare system of the future will be the definition of health, the ability to monitor changes in health status, and the ability to provide evidence-based interventions that improve health at both the population (i.e., generic) level and at the individual patient level (van der Greef et al., 2006). In recent years, a large number of publications have focused on establishing a new definition of health, thereby departing from the current static WHO definition toward a dynamic definition of health (Bircher, 2005; Lancet, 2009; Huber et al., 2011).

As illustrated in Figure 1, health can be depicted as the dynamic behavior between a physiological range, indicated by homeostasis and allostasis in normal daily life with physical, psychological, and social challenges and rhythms. The allostasis concept describes the system's response to an environmental challenge by anticipating, preparing for, and controlling the challenge (Sterling, 2012). When the challenge is over and the environment is restored to previous conditions, the system returns to its normal state. However, if the system cannot return to its healthy state, the system can enter a disease state, which can even become irreversible in an advanced stage (Ramautar et al., 2013; van der Greef et al., 2013). In addition, the “life pyramid” depicted in Figure 1 is integrated, underscoring the need to reach higher overarching network dynamics in order to develop optimal strategies for promoting health. Because health is dependent upon the environment (as discussed above), this higher level organization must be studied from a broad perspective that includes the dynamic interactions between the human body and its psychosocial environment.

The key feature of this view is that the system can restore itself via self-regulatory mechanisms, thereby maintaining health, whereas disease develops when the system loses this ability. In other words, in a disease situation, the system loses its ability to recover fully. In this situation, a strategy for promoting health is needed that differs fundamentally from the current strategy designed to simply manage disease. For this reason, preventive strategies that use pathology-based methodologies are not applicable, and we should not focus on a change in the concentration of a given molecule (for example, glucose). Thus, with type 2 diabetes, for example, we must focus on dynamic regulatory mechanisms in the system's response to a challenge (e.g., an oral glucose tolerance test). Such strategies have been developed recently using metabolomics-based strategies (Wopereis et al., 2009; van Ommen et al., 2014); however, the need still exists for a dynamic tool that can integrate, interpret, and correlate detailed information regarding regulatory mechanisms with a higher level of systems-based thinking.

Systems-based thinking has developed through systems biology in life sciences and is used to study organizations in a wider context. An important feature of systems-based thinking is its focus on relationships rather than individual variables (van der Greef et al., 2007). In such a view, the relationships between biological, psychological, social, and environmental factors—and changes in these relationships over time—are central features. By considering the complexity of these interactions, we can develop a personalized approach that is applicable in the context of the individual. This approach is centered on achieving an optimal relationship between the patient and the healthcare provider.

Metabolomics technologies are used to measure metabolite profiles in bodily fluids, thus reflecting the complex interaction between the environment and the body (van der Greef et al., 2013). To date, most system-wide measurements have been interpreted at the “medium-to-low” level of the triangle (see Figure 1) using metabolomics. Indeed, dynamic measurements of metabolites can give insight into the development of diseases and the early stages of metabolic dysfunction (Snyderman, 2012). Extremely early changes at higher levels are believed to be responsible for a shift toward disease, and detecting these changes is essential in order to develop prevention-based strategies to promote health. Indeed, measuring a system's coherence may serve as a tool for measuring the system's resilience and the individual's homeostatic and allostatic capacity.

Recent evidence suggests that recording metabolic shifts by measuring UPE-related metabolic processes in the human body can reflect the dynamics of metabolic organization (Bajpai et al., 2013; Van Wijk et al., 2014). Photobiological response (the result of chemical and/or physical changes induced by light in biological systems) and low-level biological luminescence (the production and emission of photons) are considered to be complementary manifestations of the photons' role in metabolism. Thus, the recorded photon emissions reflect the net activity of a subset of these reactions, thereby reflecting the body's current metabolic state. UPE may therefore serve as a suitable complementary tool for analyzing a biological dynamic system in combination with metabolomics technology.

UPE is present in all living organisms (van Wijk, 2014) and is low-intensity (non-thermal) light emitted from living systems without the use of an external intervention (Devaraj et al., 1997; Schwabl and Klima, 2005; Cifra and Pospíšil, 2014). Biological systems spontaneously emit a measurable number of photons within a specific range of the electromagnetic spectrum (UV and UV/VIS) as a result of normal biochemical reactions. In living systems, these photons are usually in the 300–750 nm range (Bajpai et al., 2013), depending on the system. In human tissues, the wavelength ranges from 420 to 570 nm (Wijk R. V. and Wijk E. P., 2005). The rate of photon emissions is generally on the order of 10^1^–10^3^ photons·s^−1^·cm^−2^ (Cifra and Pospíšil, 2014), and these photons originate from oxidative metabolic reactions and are therefore closely related to the rate of electronic transport in mitochondria and the generation of reactive oxygen species (ROS), reactive nitrogen species (RNS), and/or lipid peroxidation (Kobayashi et al., 1999; Cifra and Pospíšil, 2014; Pospíšil et al., 2014). When perturbed, these reactions can give rise to excessive amounts of ROS, causing damage to lipids, nucleic acids, and proteins. These damaged biomolecules can cause a loss of cellular functions, including cell signaling, immune responses, and pathways that regulate pro-inflammatory and/or anti-inflammatory processes (Van Wijk et al., 2008; Pospíšil et al., 2014), ultimately leading to cell death.

The feasibility of recording UPE as a tool for measuring dynamic changes in human health and various physiological conditions has been reported (Bajpai et al., 2013; van Wijk, 2014; Van Wijk et al., 2014) and was reviewed recently (Ives et al., 2014). For example, a wide diversity of conditions are associated with changes in the UPE profile, including aging (Sauermann et al., 1999), diurnal biological rhythms (Cohen and Popp, 1997; Wijk E. P. and Wijk R. V, 2005; Cifra et al., 2007; Van Wijk et al., 2007; Kobayashi et al., 2009), and conscious activities (Van Wijk et al., 2005, 2006, 2008). In addition, some groups have suggested that UPE properties can be used as a diagnostic tool in measuring health and disease (Usa et al., 1994; Cohen and Popp, 1998, 2003; Jung et al., 2003; Yang et al., 2004).

The technology needed to continuously monitor spontaneous UPE in the visible range in human subjects includes a sensitive photomultiplier tube (PMT) in a sealed dark environment. Such equipment has been developed and validated and is now considered a rapid, relatively inexpensive, non-invasive technology for reliably measuring UPE (Van Wijk et al., 2014).

## Combining UPE with metabolomics technology

**Figure 2:**
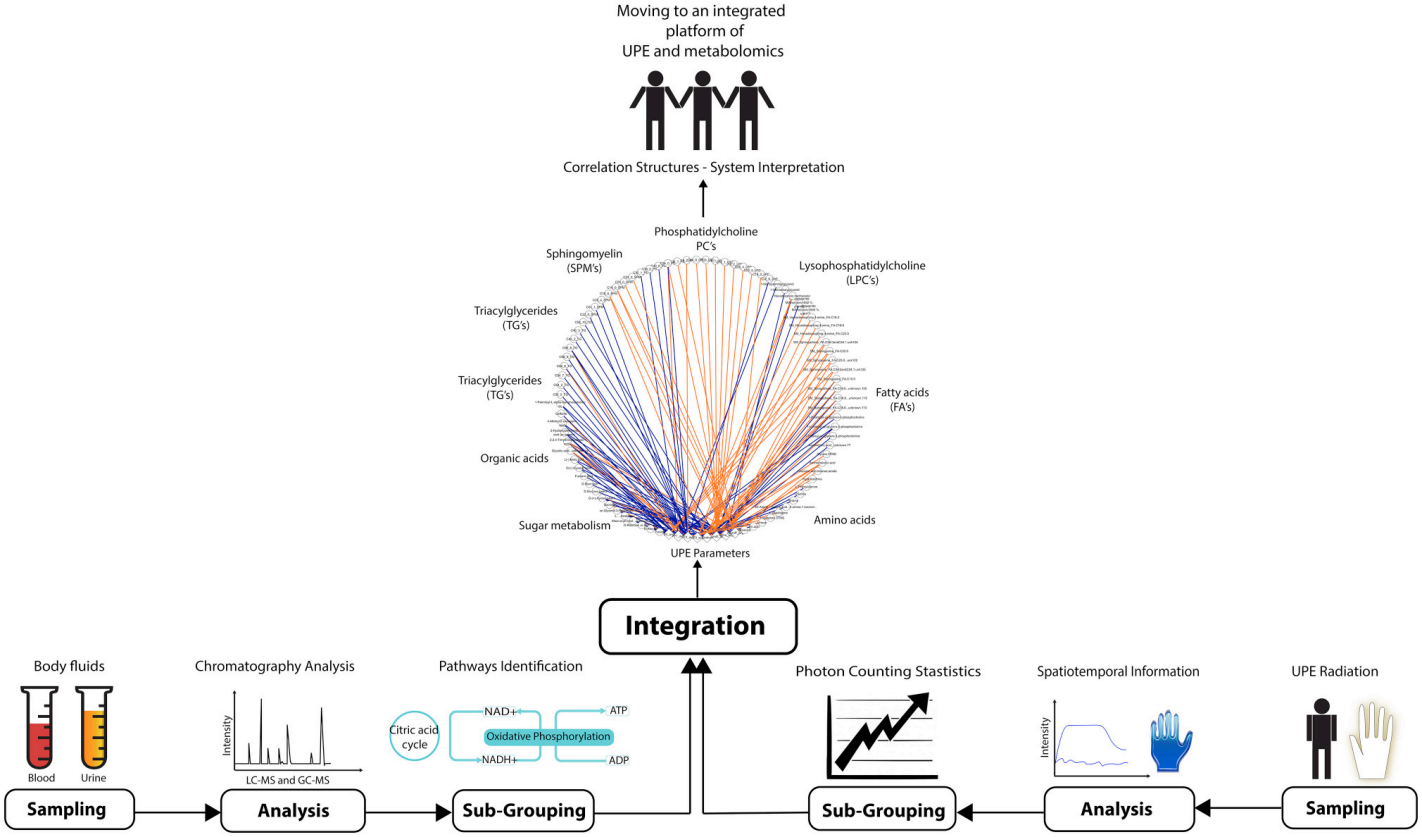
**Overview of the experimental steps required for integrating UPE and metabolomics data in order to promote health and diagnostics**. Bodily fluids (for metabolomics; left) and parts of the body (for UPE recording; right) are sampled. The samples are analyzed using chromatographic techniques (for metabolomics) and spatiotemporal analyses (for UPE). The metabolomics data are then gathered in pathways, and photon counting statistics is applied to the UPE data. Finally, the two data sets are integrated performing Spearman's rank correlations using network correlation, ultimately generating a systems-based interpretation. In this example, plasma samples of pre-diabetic subjects (44 individuals) were analyzed for the generation metabolomics data. The metabolomics study used GC-MS and LC-MS platforms to profile lipids (phosphatidylcholine, lysophosphatidylcholine, sphingomyelin, fatty acids, and triglycerides), organic acids, sugar metabolites, and amine metabolites. UPE data was acquired from the subjects' hands generating 13 parameters after applying photon count statistics. Spearman's rank correlations was calculated between UPE parameters and various classes of compounds acquired from the metabolomics analysis. The correlation was filtered using |r| > 0.3 and subsequently built the correlation network using CytoScape software (MetScape plugin). Blue lines represent negative correlations, and orange lines represent positive correlations. The study was designed and conducted by TNO (Zeist, the Netherlands). The clinical trial (https://clinicaltrials.gov/ct2/show/NCT00469287) was approved by the Medical Ethics Committee of Tilburg (METOPP).

The ability to combine and correlate UPE data with metabolomics data is an essential step toward personalized monitoring of physiological changes associated with health and disease. In life sciences, most specialized techniques generate profiles that provide a representative (i.e., generic) picture of a biological system. Both metabolomics and UPE provide an efficient readout providing valuable information regarding the dynamics of the biological systems. The challenge is to combine these two methodologies in order to link the detailed biochemistry with higher organizational information. Figure 2 illustrates the four steps that are needed: sampling, analysis, sub-grouping, and correlation. Analyzing UPE data can require robust mathematical procedures in order to study the spatial and/or temporal features of the signal, as well as to determine the photon count distributions for more sophisticated analyses (Kobayashi et al., 1998; Kobayashi and Inaba, 2000; Bajpai, 2005; Van Wijk R. et al., 2006; Bajpai et al., 2013). Finally, the two sets of data are used to build network correlations between UPE and metabolic profiles, which can then be used for individual/personalized diagnostics.

As illustrated in Figure 2, this integration approach can be exemplified in an explorative study using UPE and metabolomics data measured in pre-diabetic subjects. In this example, metabolomics data was acquired using plasma samples. Samples were analyzed using established chromatographic methodologies using GC-MS and LC-MS (Koek et al., 2006; Draisma et al., 2008; van Wietmarschen et al., 2013), followed by data processing (Xia et al., 2009). UPE data were acquired from the same subjects measuring the hands of each subject. Subsequently, derived parameters were calculated applying photon counting statistics (Van Wijk R. et al., 2006; Van Wijk et al., 2010; Bajpai et al., 2013). Correlation analysis between metabolomics and UPE data was performed using Spearman's rank correlation observing the correlation networks built in CytoScape with the MetScape plugin (Shannon et al., 2003; Gao et al., 2010). The resulting network shows correlations between the metabolomics data and the UPE data (Figure 2). Specifically, sugar metabolites and amine metabolites (which are related to energy metabolism) were positively and negatively correlated, respectively, with the UPE data (Figure 2). Lipids are known to relate to cellular signaling and energy processes and this class of compounds were also correlated with UPE parameters. The outcome might indicate an essential role in both pre-diabetes as well as the production of UPE, which is associated with energy production (Tarusov et al., 1960, 1962; Barenboǐm et al., 1969).

Overall, the integrated platform illustrates that combining UPE and metabolomics data is feasible and has high potential for both measuring specific and complex information. Specifically, integrating UPE with metabolomics contributes to our understanding of dynamic changes and provides essential insight into the underlying biochemistry, which enables to put the biochemistry information (detailed field) into a broader context and higher level of complexity. This combined approach may well be the key to realizing strategies designed to promote health.

## Concluding remarks and perspectives

Here, we indicate the potential of integrating UPE and metabolomics as a novel technology approach in order to move our healthcare system in the direction of promoting health in a proactive manner. Importantly, this integrated approach combining UPE and metabolomics can provide multi-scale information regarding key biological processes. The clear advantage of this approach is that it will improve our understanding of dynamic higher organizational levels while increasing our understanding of the underlying biochemistry at other levels. This might reveal the regulatory connection between different time scales as occurs from the cellular to the organism level.

The integrated information regarding metabolic activity and complex dynamicity could possibly further provide a rapid, robust readout of the biopsychosocial environment. In addition, combining UPE with metabolomics technology in an integrated platform for diagnosing both health and disease can provide an essential first step in the direction of systems-based thinking in personalized medicine, thereby crossing the boundaries of our current healthcare system by shifting diagnostic focus to higher organizational levels.

## Author contributions

RCRB, Jv, EV, RV conceived the manuscript. RCRB, Jv, EV, RV, and MH wrote the manuscript. All authors reviewed the manuscript and approved for publishing.

## Funding

RCRB was supported by the “Science without Borders” program of the Brazilian National Council for Scientific and Technological Development—CNPq (Conselho Nacional de Desenvolvimento Científico e Tecnológico) in the form of a research fellowship awarded for PhD training in the Netherlands (fellowship number 230827/2012-8). MH was supported by the Chinese Scholarship Council (scholarship number 20108220166).

### Conflict of interest statement

The authors declare that the research was conducted in the absence of any commercial or financial relationships that could be construed as a potential conflict of interest.
